# Adaptive Dynamics of Extortion and Compliance

**DOI:** 10.1371/journal.pone.0077886

**Published:** 2013-11-01

**Authors:** Christian Hilbe, Martin A. Nowak, Arne Traulsen

**Affiliations:** 1 Evolutionary Theory Group, Max-Planck Institute for Evolutionary Biology, Plön, Germany; 2 Program for Evolutionary Dynamics, Harvard University, Cambridge, Massachusetts, United States of America; University of Maribor, Slovenia

## Abstract

Direct reciprocity is a mechanism for the evolution of cooperation. For the iterated prisoner’s dilemma, a new class of strategies has recently been described, the so-called zero-determinant strategies. Using such a strategy, a player can unilaterally enforce a linear relationship between his own payoff and the co-player’s payoff. In particular the player may act in such a way that it becomes optimal for the co-player to cooperate unconditionally. In this way, a player can manipulate and extort his co-player, thereby ensuring that the own payoff never falls below the co-player’s payoff. However, using a compliant strategy instead, a player can also ensure that his own payoff never exceeds the co-player’s payoff. Here, we use adaptive dynamics to study when evolution leads to extortion and when it leads to compliance. We find a remarkable cyclic dynamics: in sufficiently large populations, extortioners play a transient role, helping the population to move from selfish strategies to compliance. Compliant strategies, however, can be subverted by altruists, which in turn give rise to selfish strategies. Whether cooperative strategies are favored in the long run critically depends on the size of the population; we show that cooperation is most abundant in large populations, in which case average payoffs approach the social optimum. Our results are not restricted to the case of the prisoners dilemma, but can be extended to other social dilemmas, such as the snowdrift game. Iterated social dilemmas in large populations do not lead to the evolution of strategies that aim to dominate their co-player. Instead, generosity succeeds.

## Introduction

Repeated games are among the best-studied objects in game theory, and the iterated prisoner’s dilemma has stimulated research on the evolution of cooperation for more than five decades [1]–[5]. The prisoner’s dilemma describes a social dilemma between two players, each having the choice whether to cooperate or to defect. When both cooperate, they each receive a mutual reward 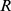, which exceeds their payoff for mutual defection, 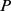. But if one player cooperates and the other defects, then the defector gets the highest payoff 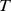, whereas the cooperator ends up with the lowest payoff 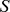. Thus, if the game is played only once (or for a known finite number of rounds), then mutual defection is the only equilibrium. However, when players cannot anticipate how often the game will be played, cooperative solutions become feasible [3], [5], [6].

Researchers from diverse disciplines have used the iterated prisoner’s dilemma to discuss the potential of direct reciprocity for the evolution of cooperation [7]–[19]. However, recently Press and Dyson [20] discovered that the infinitely repeated prisoner’s dilemma also contains strategies that allow the manipulation and extortion of opponents [21]–[25]. To show this, they first proved that there are simple strategies, which only depend on the outcome of the previous round, such that each side can enforce a linear relationship between the payoffs of the two players. More precisely, suppose player 1 applies a memory-one strategy 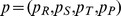, where 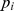 is the probability to cooperate after yielding a payoff 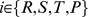 in the previous round (additionally, such a strategy needs to specify a move for the first round. However, for infinitely iterated games, the first round can often be neglected). Moreover, assume that there are three constants 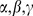 such that 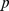 can be written as
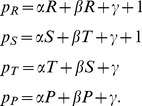
(1)


Press and Dyson [20] showed that when player 1 applies such a strategy against an opponent with arbitrary strategy 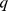, then the player’s payoff 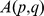 and the opponent’s payoff 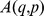 fulfill the linear relation
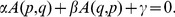
(2)


Since their proof required certain determinants to vanish, Press and Dyson called such strategies 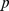 *zero-determinant strategies*. At first sight, zero-determinant strategies might seem as a mere mathematical curiosity [26]. However, their existence has several surprising consequences. Press and Dyson [20] discovered that certain zero-determinant strategies can guarantee that a player always yields at least the opponent’s payoff. They showed that by setting 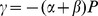, a zero-determinant strategist can enforce the relation
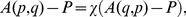
(3)where 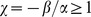 is called the extortion factor [20], [23]. Such *extortioner* strategies 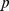 guarantee that the player’s own surplus (over the maximin value 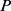) exceed’s the co-player’s surplus by a fixed percentage. In particular, when the the typical payoff relations 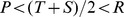 hold, the payoff of an extortioner is never below the payoff of its co-player, suggesting that extortioners would dominate any evolutionary opponent [20].

On the other hand, Stewart and Plotkin [21], [25] considered a generous counterpart to extortioners. Starting from 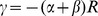, they investigated zero-determinant strategists that enforce the relation
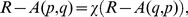
(4)where again 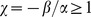. With such a generous strategy, a player can ensure that her payoff is never above the opponent’s payoff. In [23] such players are called *compliers*. Although compliant strategies seem to be too generous to succeed in competitive environments, Stewart and Plotkin [21] showed that compliers do surprisingly well in round robin tournaments, in which the compliant strategy was outperforming all other strategies (including the most prominent strategies All D, Tit for Tat, Win-Stay Lose-Shift, and an extortioner strategy). Moreover, as shown in [25], a large fraction of compliant strategies is “evolutionary robust”, meaning that no mutant with another strategy can have a selective advantage over a resident population of compliers.

Zero-determinant strategies thus have remarkable conceptual properties, but comparably little is known which of these strategies would evolve in a natural setup. It has recently been argued that extortioners are evolutionarily unstable [22]: since extortioners demand an extortionate share from any surplus, two interacting extortioners would end up with a surplus of zero. Moreover, numerical simulations indicate that zero-determinant strategies in general are disfavored by selection in sufficiently large populations [23]. However, this does not preclude certain zero-determinant strategies, such as compliers, to play an important role, as recently demonstrated by [21], [25]. To identify such important strategies, researchers have focused on particular limiting cases of zero-determinant strategies, such as extortioners, equalizers, and compliers. Moreover, to investigate the dynamics of these strategies, previous studies either had to resort to individual-based simulations, or they needed to restrict attention to a finite subset of representative strategies [22], [23], [25].

Instead, it is the aim of this study to provide an analytical framework that allows to study the evolutionary dynamics of *all* zero-determinant strategies. Constructing an analytical model for the evolutionary dynamics of the iterated prisoner’s dilemma is not straightforward. Already for simple memory-one strategies, a calculation of the resulting payoffs may become prohibitively laborious (for an example see [22]). To derive an analytical model of the dynamics, we will thus focus on an appropriate super-set of zero-determinant strategies: the set of all memory-one strategies that enforce a linear relation of the form (2), as in [25]. We show that if all players apply such strategies then the payoffs and the resulting adaptive dynamics take a remarkably simple form. In particular, we find that populations either move to the edge of compliers, or they move towards a neighborhood of unconditional defectors 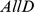. In this process, extortioners play an important role, as they can neutrally invade unconditional defectors, thereby promoting the emergence of compliance. On the other hand, altruistic strategies (such as unconditional cooperators) have the opposite effect: they can subvert a population of compliers, giving rise to the evolution of selfish strategies. Which of these strategies gets the upper hand in the long run, critically depends on the population size. While small populations favor the emergence of selfish strategies, compliance succeeds as populations become sufficiently large.

## Results

In the following, let us focus on the set of all memory-one strategies that enforce a linear relation between the payoffs of the two players. As players cannot set their own score [20], it is reasonable to consider only those strategies fulfilling Eq. (2) for which 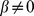 (formally this means that we exclude the strategy 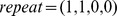 from the set of zero-determinant strategies, which is fully dependent on the initial condition). In the appendix we show that this subset of strategies is then identical to the set
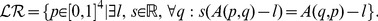
(5)


**Figure 1:**
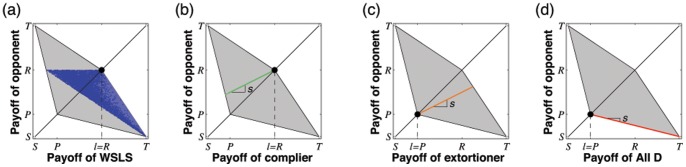
Illustration of zero-determinant strategies for an iterated prisoner’s dilemma with 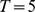, 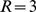, 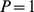 and 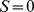. All graphs show the possible payoffs of the focal player (on the horizontal axis) and the resulting payoff for the opponent (on the vertical axis) as colored areas or lines. The colored points represent the payoff pairs for 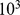 randomly chosen opponents. (a) In general, as for example when the focal player applies the win-stay lose-shift strategy 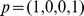, the possible payoff pairs form a convex polygon. (b) However, if the focal player applies a compliant strategy, the set of all possible payoff pairs degenerates to a line with positive slope 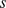, which intersects the diagonal at 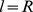. (c) An extortioner enforces payoff relations that are on a line with positive slope 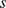, intersecting the diagonal at 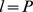. (d) The strategy 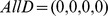 enforces a linear relation between the payoffs of the two players although 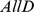 is not a zero-determinant strategy for the given parameters, as described in the Methods section.

Instead of the three parameters 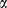, 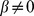 and 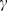, this specification only requires two free parameters, 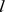 and 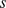. Both parameters allow an intuitive interpretation (see Figure 1). The parameter 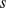 gives the correlation between both players’ payoffs. A factor 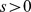 means that a player enforces a positive linear relation between the payoffs, whereas for 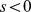, the payoffs obey a negative linear relation. The parameter 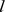, on the other hand, can be considered as the payoff that a player would get against himself (see Figure 1). We thus call the parameter 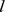 the *baseline payoff*, and we refer to 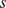 as the *slope* of an 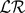
–strategy (in fact, the slope 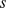 is just the inverse of the extortion factor 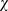).

We consider an iterated prisoner’s dilemma and make the common assumption that the payoffs of the one-shot game fulfill the relation 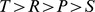, and 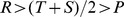, such that mutual cooperation is the best outcome and mutual defection is the worst outcome. As payoffs then need to be in the interval 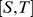, and because memory-one strategies need to consist of four probabilities, there are restrictions on the linear relations that a player can enforce. In the Methods section, we show that a pair (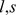) is enforceable if
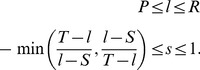
(6)


For example, the set of extortioners corresponds to the set of pairs (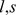) with 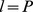 and 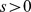. The set of compliers is given by those memory-one strategies for which 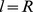 and 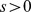. In the following, we study the evolution of zero-determinant strategies by considering the dynamics on the (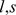)-plane. That is, we assume that each player determines an enforceable pair 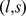 and then picks a 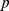 from the corresponding class of 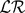-strategies. Depending on the player’s performance in the game, the enforceable pair 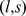 may then be adopted by others, a process that we will describe with adaptive dynamics and individual-based simulations.

### Adaptive Dynamics in Infinite Populations

In order to derive the adaptive dynamics on the 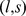
–plane, we first have to calculate the payoffs for each player. While the payoff function for general memory-one strategies is highly non-trivial, these calculations become straightforward for 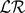-strategies. Suppose a player wants to enforce the linear relation (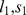) by choosing an appropriate 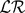-strategy 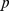, whereas the co-player enforces the pair (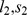) by choosing 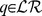. Then the payoffs are implicitly given by
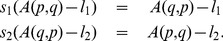
(7)


From this, we recover the result that a player can set the co-player’s score to a fixed value [20], [27]: by choosing 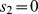, player 2 can guarantee that the first player’s payoff is 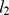 (i.e., the set of so-called equalizers corresponds to all enforceable pairs (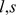) with 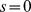).

Excluding the two non-generic cases that both players enforce the most extreme payoff relations (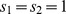 or 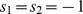), this system of two linear equations has a unique solution for the payoffs
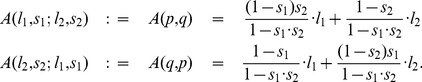
(8)


It follows that if both players have the same baseline payoff, 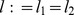, then their payoff will be 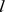, irrespective of their choice of the slopes 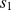 and 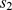. In particular, the payoff of a homogeneous 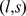-population is 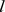. As a consequence, if we consider homogeneous populations, and if we assume that the populations move towards the direction where mutants have the highest invasion fitness, then the resulting adaptive dynamics [28]–[30] is given by
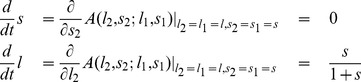
(9)


**Figure 2:**
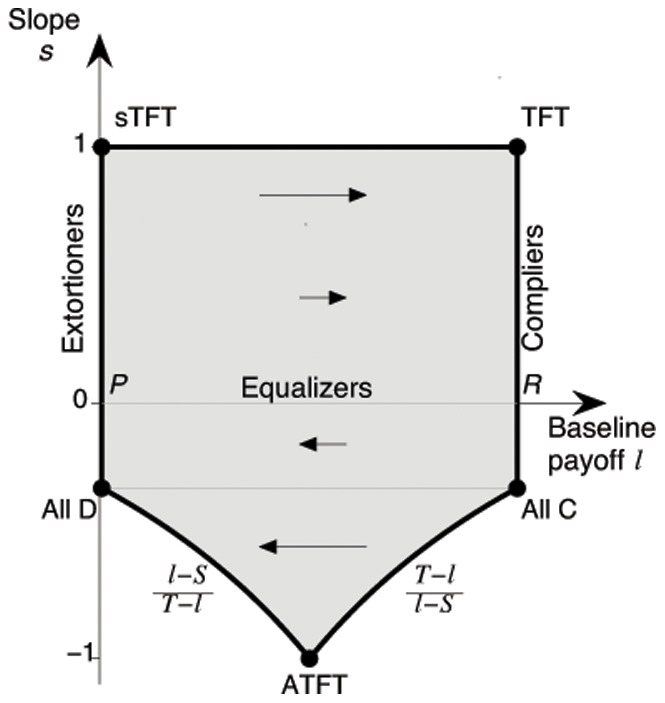
Adaptive dynamics in the (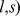-plane. The grey-shaded state space represents the set of all enforceable linear relations that fulfill the inequalities (6). The corners of this state space consist of the payoff relations (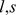) that correspond to the five strategies Always Cooperate (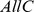), Tit-for-Tat (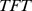, which starts with cooperation, and then repeats the opponent’s previous move), Suspicious Tit-for-Tat (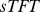, which starts with defection and then repeats the opponent’s previous move), Always Defect (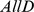), and an Anti-Tit-for-Tat strategy (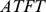, which always plays the opposite of the opponent’s previous move). Three special subsets of this state space are of particular interest: (i) Extortioners are strategies for which 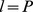 and 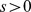. (ii) Equalizers are strategies with 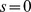 (iii) Compliers correspond to the edge 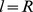 and 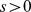. The grey line between 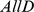 and 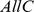 corresponds to the set of linear relationships that can be enforced with unconditional strategies (in particular it follows that all unconditional strategies enforce linear relationships with a negative slope, see Methods section). The adaptive dynamics for this system is surprisingly simple: orbits are parallel to the 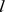-axis; for 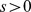, they converge towards the edge of compliers, whereas for 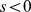, they converge towards the left boundary of the state space. Parameters: 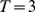, 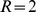, 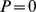, 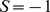.

The first equation implies that the slope 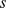 remains constant under adaptive dynamics. Nevertheless, the initial value of 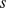 determines the eventual fate of the population: if individuals enforce a positive correlation between payoffs (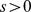), then the baseline payoff 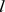 increases over time. Eventually, such a population will thus yield the maximum payoff 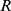, i.e. the population converges to the edge of compliers, see Fig. 2. On the other hand, for 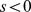 the population payoffs 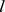 decrease over time, and the dynamics leads to strategies in the neighborhood of 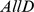. Interestingly, although extortioners always outcompete their direct opponent, the edge of extortioners is unstable, as illustrated in Fig. 2. Along this edge, mutants with higher baseline payoff 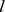 can invade. By giving in the extortioners’ claim, they are able to yield a payoff that exceeds the payoff 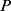 that extortioners get against themselves. However, this argument rests on the assumption of an infinite population, such that the probability for an extortioner to interact with a rare, but profitable mutant is zero. In the following section, we therefore extend our analysis to finite populations.

### Adaptive Dynamics in Finite Populations

Extortioners play a more prominent role in finite populations [23], where pairwise payoff advantages have a stronger effect (see also [14], [31]). This is most intuitive when the population only consists of two individuals; since extortioners outperform their direct co-player by definition, extortion is expected to spread. These observations suggest that a given extortionate strategy can be stable as long as the population size is below some critical threshold. To calculate this threshold analytically, let us consider a homogeneous population of size 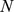 that enforces the pair 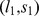. From time to time, a player may mutate to a different enforceable pair (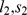). If mutation (or exploration) events are sufficiently rare, the strategy of the mutant goes extinct, or fixates, before the next mutation occurs [32], [33]. In this case, the fixation probability 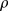 is the decisive quantity for the evolutionary dynamics. It can be shown that such a process can be described with a modified form of the adaptive dynamics equation; instead of asserting that homogenous populations move towards the direction where mutants have the highest invasion fitness, it is assumed that the population moves towards the direction where mutants have the highest fixation probability. In Imhof and Nowak [34] it is shown that this direction can be found by calculating the adaptive dynamics for a slightly perturbed payoff matrix (called the effective payoff matrix, or modified payoff matrix, see [35], [36]),
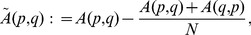
(10)


The first correction term, 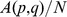 means that individuals cannot play against themselves, whereas the second correction term 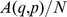 corresponds to the competition effect in finite populations. In our case, the adaptive dynamics for finite populations becomes
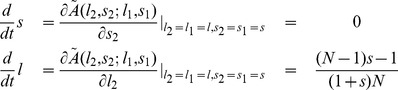
(11)


Remarkably, the slope 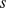 remains invariant for all population sizes. However, the dynamics for the baseline payoff 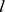 changes for small 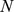: in the extreme case of 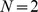, all trajectories in the interior of the state space lead to the lowest possible population payoff. For 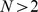, a bistable situation emerges: if the value of 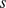 in the initial population exceeds 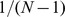, then the population moves towards the edge of compliers (with 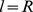), whereas for smaller values of 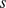 populations move towards a non-cooperative equilibrium (with 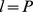). Therefore, larger populations promote the evolution of cooperative behaviors, and in the limit of infinitely large populations, 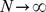, we recover the original adaptive dynamics (9). The dynamical equations (11) also imply that a given extortionate strategy can only be stable if 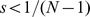, or equivalently if the strategy’s extortion factor 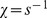 fulfills 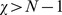. Thus, to be stable in a finite population, extortioners need to be sufficiently demanding (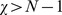), whereas compliers must not be too generous (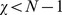).

**Figure 3:**
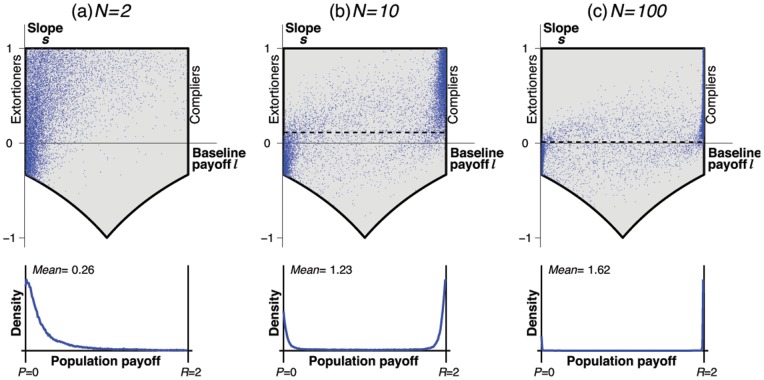
Stochastic dynamics for different population sizes. We consider a homogeneous population of size 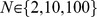. Once a mutation occurs, the mutant strategy either takes over the whole population (with probability 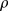), or goes extinct before the next mutation arises. This leads to a sequence of residents in the state space, which is shown in the upper three graphs (the dashed line corresponds to the threshold 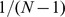). The lower three graphs give the distribution of the resulting payoffs in the population. (a) In the extreme case of 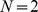, most players enforce a strategy with baseline payoff 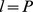. In particular, extortion strategies can persist. (b) As population size increases, a bistable situation emerges: the population clusters along the edges with 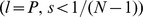 and 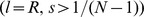. (c) For large population sizes, this implies that the edge of compliers is (neutrally) stable, whereas the edge of extortioners is unstable. As a consequence, mean payoffs increase with population size. The figure shows simulation runs for 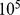 residents for a prisoner’s dilemma with 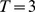, 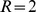, 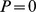, 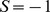. New mutant strategies are randomly drawn from a Gaussian distribution around the parent strategy (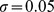). The invasion probability 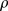 of a mutant is calculated as 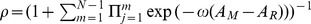, where 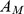 and 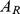 are the respective payoffs of mutants and residents, and where 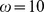 is the strength of selection.

In order to confirm these predictions, we have simulated the dynamics in finite populations for a pairwise comparison process, where the probability to switch to the role model’s strategy is given by a Fermi function [37], [38]. We assume that mutations follow Gaussian distributions around 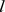 and 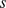 and focus on the distribution of strategies and on the distribution of payoffs. For 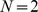 we find that the population clusters around the edge of low population payoffs (see Fig. 3a), and the density function for the payoffs has a single peak at 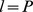. Increasing the population size has a two-fold effect (Fig. 3b and 3c). First, compliant strategies with 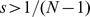 become stable, such that the density function of the population payoffs has a second peak at 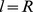. Second, increasing the population size reduces the stochastic noise; as a consequence almost all the mass is concentrated around the two peaks 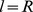 and 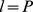. As predicted by adaptive dynamics, and in line with previous results [23], larger populations exhibit larger payoffs. For example, payoffs for a population size 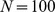 exceed the payoffs for 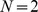 by more than a factor of six.

**Figure 4:**
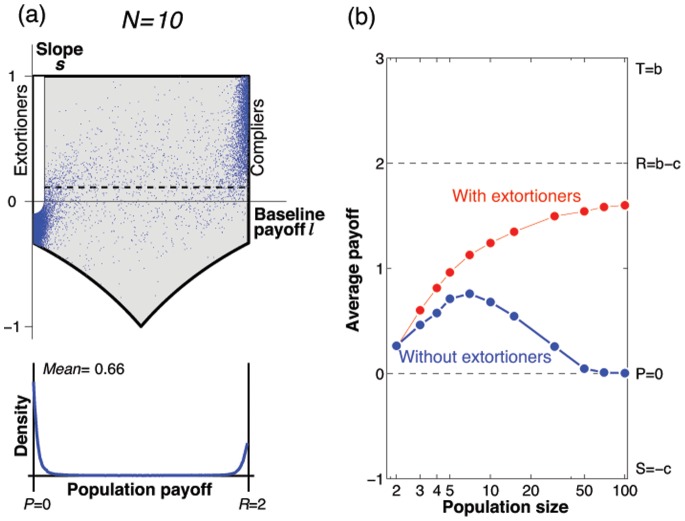
Extortioners facilitate cooperation. In order to study the impact of extortioners on the evolutionary dynamics, we have excluded all mutant strategies that are 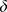-close to the set of extortioners. (a) For the simulations we have used 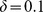, represented by the white area in the upper left corner of the panel. (b) As a result, we find for all population sizes 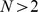 that the removal of extortioners decreases the average payoff. This decrease is particularly dramatic in large populations, 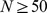. Parameters are the same as in Fig. 3.

Although extortioners seem to apply a fully selfish strategy, they are important as they can act as a catalyst for cooperation, by helping the population to escape from states with low payoffs [23]. Our adaptive dynamics formalism allows us to give an intuitive explanation for this effect: under a local mutation scheme, a population of 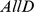 players can only be invaded by neutral drift, by moving along the vertical line of strategies with 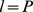. For cooperative strategies to have a selective advantage, the new resident population needs to have a positive slope 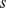 (i.e., only when the new resident applies an extortionate strategy, cooperation can evolve). In order to confirm this catalytic effect of extortionate strategies, we have removed a 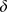-neighborhood around the edge of extortioners from the set of enforceable pairs (see Fig. 4a; in [34] this method is called a knock-out experiment). That is, only those mutants are permitted that are sufficiently different from extortioners. The result is surprising: although extortioners are defined as strategies with the lowest payoff against themselves, their exclusion reduces the average payoff of the population for all population sizes 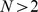 (Fig. 4b). This effect is especially pronounced in larger populations; for 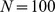, Fig. 4b indicates that it is almost impossible to reach a cooperative regime without extortioners.

**Figure 5:**
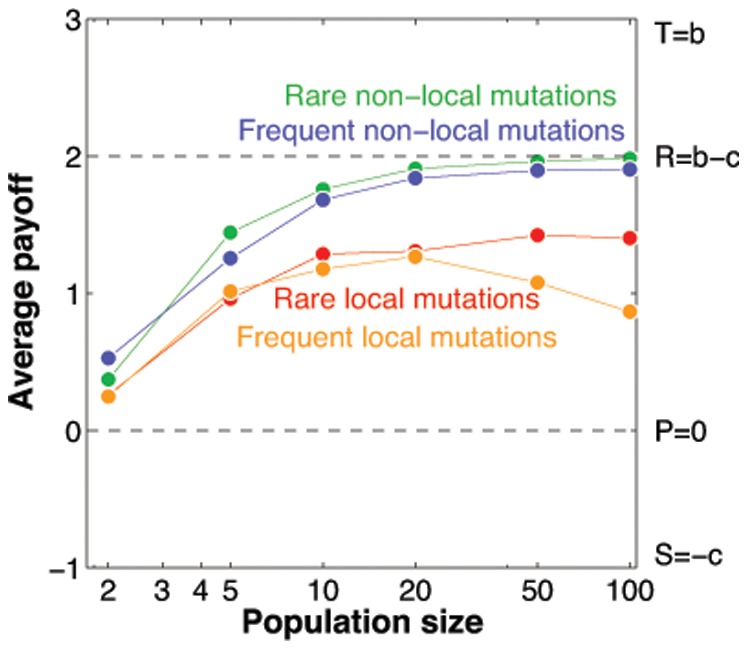
Average payoffs for the four different mutation treatments. In rare-mutation treatments, the mutation rate is set to 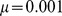, whereas in frequent-mutation treatments the mutation rate is 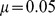. Local mutations are randomly drawn from a Gaussian distribution around the parent strategy, non-local mutations are randomly drawn from the entire state space. The rare local mutations correspond to the previous simulations in Figs. 3 and 4. All other parameters are the same as before.

So far, we have assumed that a mutant’s strategy is close to the parent’s strategy (which allowed us to use derivatives to approximate the dynamics), and that mutations are rare (which allowed us to focus on games between a resident and one mutant strategy). Let us now weaken these assumptions and numerically explore the impact of non-local mutations, and of different mutation rates, respectively. In Fig. 5, we distinguish four simulations, according to whether the mutation rate is high or low (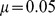 vs. 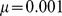), and whether mutations occur on a local or on a global level (mutant strategies are drawn from a normal distribution around the parent’s strategy, vs. mutant strategies are uniformly distributed over the set of enforceable pairs). These simulations indicate that all treatments follow the same pattern: average payoffs are close to the minimum 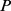 in small populations, and they increase with population size. However, there is a clear difference between treatments with local mutations and treatments with non-local mutations. If mutations are local, populations can be trapped in regions with a low payoff for a considerable time, although distant mutant strategies would offer an immediate escape. For example, we have seen that any strategy of the form 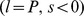 forms a stable fixed point of the adaptive dynamics. However, once we allow mutants to adopt any strategy of the state space, mutants with 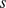 close to one and 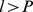 can easily invade (in fact, in Stewart and Plotkin [25] it is shown that in sufficiently large populations, compliant strategies with 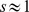 can replace any noncooperative zero-determinant strategy). Overall, non-local mutations thus lead to a shift of the invariant distribution towards more cooperative strategies.

## Discussion

The set of zero-determinant strategies exhibits a fascinating variety of possible behaviors, ranging from extortioners to compliant strategies, and from selfish strategies to altruists. To evaluate the evolutionary relevance of these different possible behaviors, previous studies focused on particular subsets. Adami and Hintze [22] demonstrated that neither extortioners nor equalizers are evolutionarily stable, and Hilbe et. al. [23] confirmed numerically that these two subsets are only favored by selection if the population is sufficiently small. In contrast, as shown by Stewart and Plotkin [25], large population sizes favor the emergence of compliant strategies, which are evolutionary robust (they can only be invaded by neutral drift), and which in turn are quite successful in invading other strategies. However, this focus on specific subsets of zero-determinant strategies comes at the risk of neglecting other important subsets. Thus, here we have systematically explored the space of all zero-determinant strategies.

To this end, we have derived the adaptive dynamics for all strategies that enforce a linear relation between the payoffs of the two players. This set of strategies includes all zero-determinant strategies [20] and all unconditional strategies such as 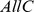 or 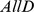 (see Methods section), but not all memory-one strategies (for example, it does not contain the win-stay lose-shift rule depicted in Figure 1a). The focus on this strategy space allows us to describe the evolutionary dynamics with an analytically tractable model. The resulting dynamics in large populations is bistable and the state space contains two neutrally stable sets. When the initial population enforces a positive relation between payoffs (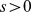), the population is most likely to end up at the edge of compliers. This subset of strategies shares the following three properties: (i) compliers enforce a linear relation between the payoffs of the two players, (ii) a population of compliers yields the maximum possible payoff 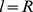, and (iii) compliers play a best response to themselves (no strategy can yield a payoff higher than 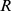 when playing against a complier, see also [24] for a characterization of such strategies). However, compliers have one shortcoming: they can be neutrally invaded by altruistic strategies (strategies that accept a decrease of their own payoff to increase the opponent’s payoff, such as 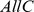 with 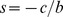). Such altruistic strategies give rise to selfish behaviors, leading the population to a neighborhood of 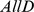. To escape from that neighborhood, extortioners play an important role [23]: they can invade 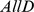 by neutral drift and promote the emergence of compliant strategies. Thus, the route from cooperation to defection goes via altruism, whereas the route from defection to cooperation goes via extortion.

**Figure 6:**
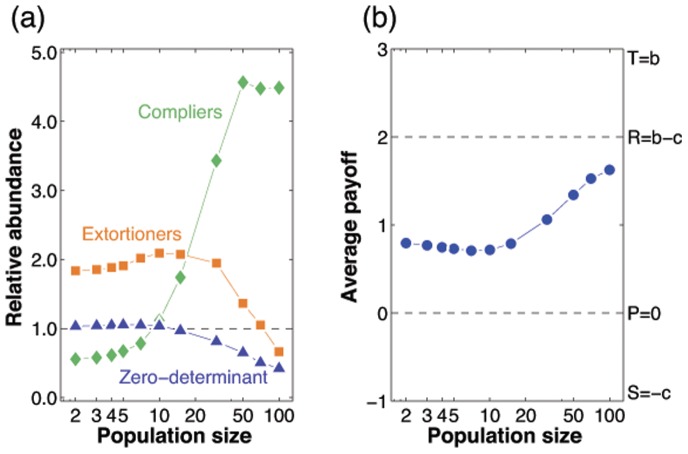
Statistics for the stochastic dynamics on the space of all memory-one strategies. Instead of taking the enforceable pairs (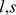) as the evolving traits, we consider the adaptive dynamics on the space of memory-one strategies 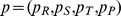, see also [23], [25]. (a) To assess the impact of zero-determinant strategies, extortioners, and compliers, we record how often the evolving population is in a 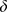-neighborhood of these strategy sets, and compare this to their expected abundance in a neutral process. A given strategy set is thus favored by selection if its relative abundance exceeds one. Our simulations indicate that in small populations extortioners are favored by selection, whereas in large populations compliers are favored. (b) As a consequence, average payoffs increase with population size. Simulations are run for a sequence of 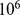 mutants. We assume that mutant strategies are uniformly distributed over the space of memory-one strategies, and use the parameters 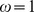 and 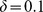. The other parameters of the evolutionary process are the same as in the previous figures.

It is natural to ask which of these dynamical results on the space of all zero-determinant strategies are robust when we consider evolution in more general strategy spaces, such as memory-one strategies, or strategies encoded by a finite automaton (see, for example, [5]). Further simulations suggest that our results hold more generally: for Fig. 6 we consider the adaptive dynamics on the space of all memory-one strategies (similar simulations are also presented in [23], [25]). The numerical results confirm our analytical predictions based on the adaptive dynamics framework: extortioners are strongest in small populations, whereas compliers succeed in large populations. Note, however, that zero-determinant strategists in general are disfavored by selection as the population size increases. In fact, as our analysis suggests, a large proportion of zero-determinant strategies only play a transient role in the evolutionary dynamics. For most of the time, the population applies a strategy that is close to one of the boundaries 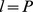 and 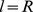, whereas interior states are hardly visited. The dynamics is centered around the edge of selfish strategies and extortioners, and around the edge of compliers and altruists, whereas the evolutionary importance of other zero-determinant strategies seems negligible.

Our results on the adaptive dynamics of zero-determinant strategies resemble the results for the evolution of reactive strategies (i.e., memory-one strategies with 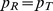 and 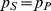, [5], [28], [34]). In both models, there are two regimes. There is a *cooperation rewarding zone* where populations evolve towards an edge of fully cooperative strategies (the edge of compliers, or the edge between tit-for-tat and generous tit-for-tat, respectively). Outside of this cooperation rewarding zone, populations move towards lower population payoffs (ending up at a neighborhood of 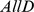). These similarities are not a mere coincidence. Instead, for games with equal gains from switching (when 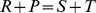), every reactive strategy is a zero-determinant strategy [23] and thus reactive strategies form a subset of 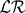. Conversely, we show in the Methods section that any enforceable payoff relation (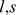) can be enforced by a reactive strategy in this case. Thus, for games with equal gains from switching, the space 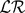 is essentially equivalent to the space of reactive strategies.

**Figure 7:**
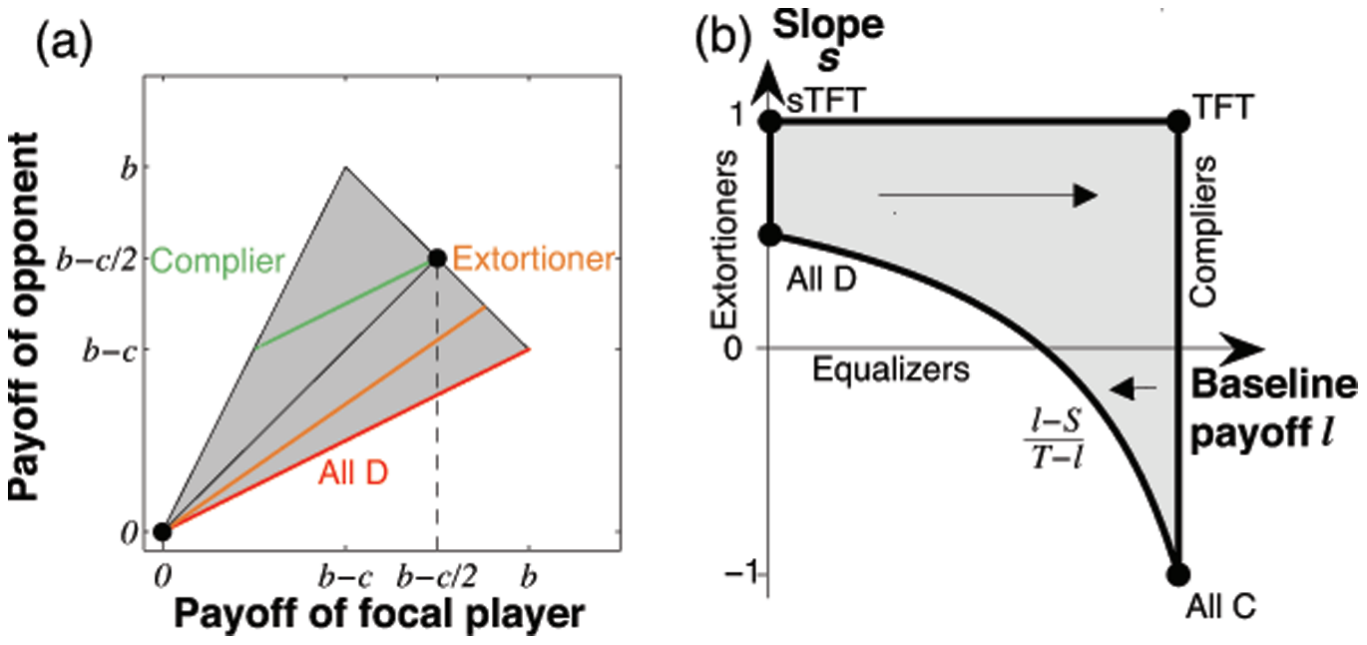
Zero-determinant strategies for the iterated snowdrift game (with 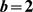, 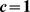, and 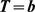, 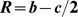, 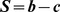, 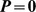). (a) The grey shaded area gives the space of feasible payoff pairs in the snowdrift game. The three colored lines give three examples of possible payoff combinations if the focal player uses a strategy that enforces a linear relation between payoffs. Unlike in the iterated prisoner’s dilemma, the slope of 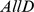 is positive, 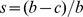. (b) The grey-shaded area depicts the space of possible combinations of baseline payoff 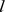 and slopes 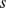 that are enforceable in the snowdrift game. A comparison with Fig. 2 shows that the state space differs considerably from the state space of a prisoner’s dilemma game. However, the qualitative dynamics within the state space remains unchanged.

Throughout this manuscript, we have focused on the dynamics of an iterated prisoner’s dilemma. However, only a few of our results actually depend on the characteristic order of payoffs, 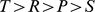. In fact, the only result specific to the prisoner’s dilemma concerns the characterization of enforceable (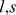) pairs in Eq. (6). For games that are different from the prisoner’s dilemma, the geometry of the state space may thus be different, but the dynamics on the respective state space remains unchanged. In Figure 7, we illustrate this observation by considering the dynamics of an iterated snowdrift game (which is defined by the payoff relations 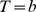, 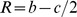, 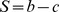, 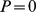 with 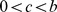 such that 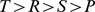, see [39], [40]). For snowdrift games we observe that only a subset of extortionate strategies is feasible [41]: extortionate strategies with 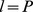 need to fulfill the requirement 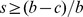 (i.e. the maximum extortion factor is 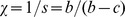). Moreover, only strategies that yield a baseline payoff higher than 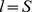 can enforce a payoff relation with negative slope, 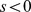. As a consequence, any sufficiently large initial population that yields a payoff less than 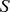 against itself can be replaced by more cooperative mutant strategies with higher baseline payoffs. As in the prisoner’s dilemma, this dynamics leads to the edge of compliers, which can only be left by neutral invasion of altruists.

Similar results may be feasible for social dilemmas with a continuous action space, as for example considered in [42]–[46]. However, transferring our findings to the continuous case is not straightforward. First, the existing literature on zero-determinant strategies exclusively deals with games where the players can only choose among two actions (either to cooperate or to defect), and it is not obvious how the corresponding proofs can be generalized to iterated games with continuous action spaces. Moreover, even if continuous games admit zero-determinant strategies, one may wonder which linear relations (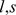) these strategies can enforce. Is there an upper bound on the extortion factor? Which payoffs can be enforced by an equalizer strategy? The answers to these questions are likely to depend on specific details of the benefit and cost function, representing an interesting topic for future research.

Our results confirm that extortionate behaviors can only prevail in small populations. In large populations, the evolutionary steady state is increasingly biased in favor of cooperative strategies. This may come as a surprise, as it has been shown that intermediate population sizes are optimal for the fixation of rare cooperative mutants in a population of defectors [14]. However, compliant strategies do not need to invade defectors directly. Instead, in sufficiently large populations extortioners always provide an escape path to leave non-cooperative populations. More importantly, once compliant strategies are common, they are evolutionary robust [25], with the neutral invasion of overly altruistic strategies as their only weak spot. Overall, compliance succeeds.

## Methods

### The Geometry of the State Space

Let us first show that the set of all strategies that fulfill condition (2) coincides with the set 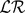, as defined by (5). If we multiply the condition
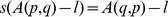
(12)with some 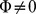, then we can relate (2) and (5) by the following transform of coordinates



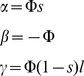
(13)It then follows from (1) that a zero-determinant strategy 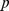 enforces the pair (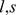) if and only if there is a 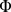 such that 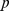 has the form
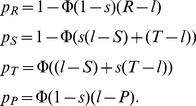
(14)


Since all entries 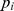 need to be in the interval 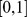, there are restrictions on the pairs (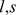) that can be enforced by zero-determinant strategies. For the parameters of the prisoner’s dilemma, it follows by 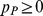 and 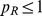 that baseline payoffs 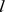 need to fulfill the condition 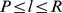. Again because 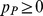 and 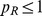, we may then conclude that 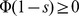. As a consequence, the requirement 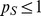 yields 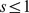 and 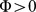. Then 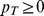 leads to the restriction 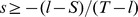, whereas 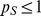 implies 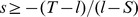. In summary, we conclude that for all pairs (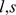) that fulfill
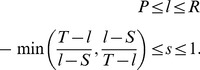
(15)there is a corresponding zero-determinant strategy 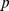 of the form (14) such that 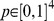 (we only have to choose a 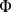 that is sufficiently small). Conversely, the linear relations 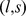 that can be enforced by zero-determinant strategies are in fact all possible linear relations that can be enforced in an iterated prisoner’s dilemma with 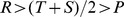. To see this, we note that for any memory-one strategy 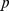 we have:

1. The payoff pair 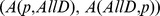 is on the line between 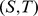 and 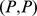, whereas
2. the payoff pair 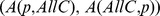 is on the line between 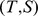 and 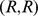.

Thus, any linear payoff relation (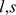) enforced by some 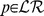 connects the line segment between 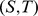 and 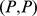 with the line segment between 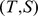 and 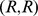 (see also Figs. 1b–1d). A straightforward computation verifies that any such linear payoff relation (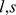) needs to meet the conditions (15).

The set 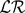 is a proper super set of the zero-determinant strategies. For example, the strategy 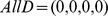 is not a zero-determinant strategy in the general prisoner’s dilemma (it is only a zero-determinant strategy in games with equal gains from switching, i.e. when 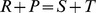). However, 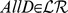 holds true in all prisoner’s dilemma games. In fact, every unconditional strategy 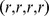 is an element of 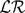, with parameters
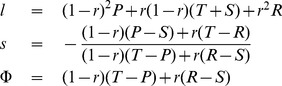
(16)


In particular, it follows that unconditional strategies can only enforce linear payoff relations with negative slopes 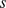. As previously suggested, these values of 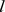 and 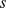 satisfy the inequalities (15) for all 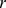; any linear relation (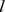,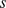) that can be enforced by an unconditional strategy can also be enforced by a zero-determinant strategy.

Given a triplet (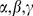), the corresponding zero-determinant strategy 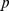 is uniquely determined by (1). However, for a given pair (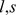) there will generally be many zero-determinant strategies 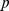 that enforce the corresponding linear relationship in (5) - one for every 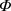 in (14). We call two strategies 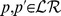 equivalent, and write 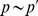, if they give rise to the same pair (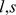). To study the evolutionary dynamics of 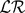-strategies, we consider the dynamics on the space of equivalence classes 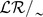. That is, we assume that each player determines a pair (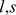) and then picks a 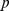 from the corresponding class of 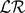-strategies. The dynamics is well-defined in the sense that the adaptive dynamics does not depend on the choice of the class representative 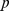.

### 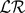-strategies Versus Reactive Strategies

When payoffs fulfill equal gains from switching, 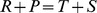, we can choose 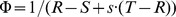 such that the zero-determinant strategies according to Eqs. (14) are given by
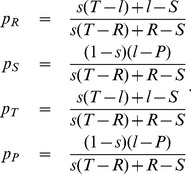
(17)


In particular, 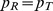 and 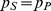, i.e. all resulting zero-determinant strategies are reactive strategies. For such reactive strategies it follows that for 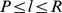 the conditions 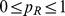 and 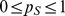 are equivalent to the conditions
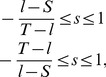
(18)respectively. From this, we conclude that for games with equal gains from switching, all payoff relations 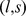 that can be enforced by zero-determinant strategies (given by the conditions (15)) can already be enforced by reactive strategies.
